# Effect of MB Value and Proportion of Reclaimed Powder on Mechanical Strength and Equivalent Cement Content of Cement-Stabilized Macadam

**DOI:** 10.3390/ma18245686

**Published:** 2025-12-18

**Authors:** Ouyang Lou, Junhao Li, Huaiping Xiao, Yingjun Jiang, Jiangang Xu

**Affiliations:** 1 Jinhua Transportation Investment Construction Engineering Investment Management Co., Ltd., Jinhua 321000, China; 13133119780@163.com; 2 Key Laboratory for Special Area Highway Engineering of Ministry of Education, Chang’an University, Xi’an 710064, China; 2024221252@chd.edu.cn (J.L.); 2022121202@chd.edu.cn (J.X.); 3 China First Highway Engineering Co., Ltd., Beijing 100024, China; othinusmail@163.com

**Keywords:** cement-stabilized macadam, reclaimed powder, mechanical strength, MB value, reclaimed powder proportion, equivalent cement content

## Abstract

The fine clay content in reclaimed powder significantly influences the mechanical properties of cement-based materials. To promote the resource utilization of reclaimed powder in road engineering, using the methylene blue (MB) value as an indicator to evaluate the fine clay content of reclaimed powder, the influence of the MB value and proportion of reclaimed powder on the mechanical strength of cement-stabilized macadam was analyzed; fitting equations for the relationship between reclaimed powder proportion and mechanical strength were constructed; the required MB value and optimal proportion of reclaimed powder were clarified; the impact of MB value variation on mechanical strength under optimal proportion conditions was evaluated; and with mechanical strength consistency as the principle, equivalence analysis between reclaimed powder proportion and cement content was conducted. The results indicate that when the MB value of reclaimed powder is less than 5.0 g/kg, the MB value has no obvious influence on the mechanical strength of cement-stabilized macadam. With increasing reclaimed powder content, both the compressive strength and splitting tensile strength of cement-stabilized macadam first increase and then decrease, reaching peak values at reclaimed powder contents of 3.0–4.0% and 5.0–5.5%, respectively. As cement content increases, the strength-enhancing effect of reclaimed powder weakens. The MB value of reclaimed powder should be less than 5.0 g/kg with a content of 4%. When cement content is 3–4%, based on mechanical strength equivalence, a reclaimed powder content of 4% can replace at least 0.4–0.5% of cement content.

## 1. Introduction

Reclaimed powder refers to fine dust with a particle size of less than 0.075 mm collected by bag filters during the production of asphalt mixtures. According to statistics, approximately 50–70 kg of reclaimed powder is generated per ton of asphalt mixture produced, and the annual output of reclaimed powder nationwide reaches tens of millions of tons. Currently, the main disposal methods are open-air stacking and landfilling, which not only occupy a large amount of land but also incur high disposal costs, exerting significant negative impacts on the urban environment and economy. However, in reality, studies by Esquinas et al. [1] and Valentin et al. [2] have proven that reclaimed powder possesses considerable reuse value. Its application in civil engineering materials can yield remarkable engineering and environmental benefits.

The main component of reclaimed powder is stone powder, which may be accompanied by a small amount of mud powder (related to the cleanliness of raw materials). The main mineral components of stone powder (CaCO_3_, SiO_2_) can undergo secondary reactions with Ca(OH)_2_ (a hydration product of cement) in an alkaline environment to form calcium silicate hydrate (C-S-H) gel with cementitious properties [3,4,5]. Meanwhile, its ultra-fine particle size can effectively fill the gaps in the aggregate skeleton, thereby improving the matrix strength and durability [6,7]. Studies by Umar, M et al. [8], and Nair et al. [9] have indicated that the use of stone powder as an admixture in cement-based materials is feasible, and both workability and mechanical properties can meet design indicators and application requirements. Research by Joyce et al. [10], Sundaralingam et al. [11], and Cohen et al. [12] has shown that an appropriate amount of stone powder can improve the mechanical properties of cement concrete. Chitkeshwar et al. [13] proposed that replacing 25% of river sand with stone powder can significantly enhance the compressive strength of concrete; some studies have directly replaced cement with stone powder in equal quantities. Jain et al. [7] pointed out that the compressive strength of concrete with 20% of cement replaced by stone powder (by mass) is higher than that of plain concrete, and Alizada et al. [14] also achieved positive results when applying stone powder to ultra-high-performance concrete. Additionally, studies by Nakayenga et al. [15] and Pakkiyacha et al. [16] have indicated that the application of stone powder in concrete can significantly reduce carbon emissions. Furthermore, stone powder has also been applied in other cement-based materials such as metakaolin-based geopolymers and cement-stabilized lateritic soil in existing research [5,17]. It can be seen from this that the application of reclaimed powder in cement-based materials is an important means for its large-scale resource utilization. As a commonly used cement-based material for high-grade highways in China, cement-stabilized macadam exhibits characteristics such as high strength, excellent integrity, good water stability, and frost resistance. The application of reclaimed powder in cement-stabilized macadam holds broad prospects and significant engineering value.

However, the practical application of reclaimed powder faces major challenges, mainly due to the small amount of mud powder it contains, which can degrade the performance of cement-based materials [18,19], leading to unstable performance of cement-based materials. Li et al. [20] pointed out that the addition of mud powder has a negative impact on the workability of mortar and concrete; mud powder can wrap around cementitious material particles or aggregate interfaces, hindering the cement hydration process and the development of interface bonding strength and degrading the mechanical properties of cement mortar. Zhao et al. [21] noted that mud powder itself has basically no cementitious activity, and its excessive presence can damage the aggregate skeleton and increase the risk of plastic deformation. It can be seen that the key to the resource utilization of reclaimed powder lies in revealing the influence law of mud powder on the performance of cement-based materials and controlling its negative impacts. However, existing studies often ignore the influence of mud powder characteristics on the performance of cement-based materials, mainly due to the lack of indicators effectively characterizing the mud powder content in reclaimed powder. In China’s engineering construction standards, such as Sand for Construction (GB/T 14684-2022) [22] and Concrete for Railway Construction (TB/T 3275-2018) [23], the methylene blue (MB) value is used to characterize the clay content in manufactured sand– and natural sand. Existing studies have confirmed [4,24,25] that the MB value has a significant linear positive correlation with the mud powder content. The mud powder in reclaimed powder and that in manufactured sand (or natural sand) both originate from natural clay in sand and stone raw materials and are highly homologous. Therefore, it is practically feasible to evaluate the clay content of reclaimed powder using the MB value.

Therefore, this study investigates the influence laws of the MB value and content of reclaimed powder on the mechanical strength of cement-stabilized macadam, establishes fitting equations for the mechanical strength with respect to the reclaimed powder content, proposes the required MB value and recommended content of reclaimed powder, explores the influence amplitude of the MB value of reclaimed powder on mechanical strength under the optimal content, conducts an equivalence analysis between reclaimed powder and cement content, and puts forward the replaceable cement content. This study is conducive to improving the resource utilization rate of reclaimed powder and provides a reference basis for the application of reclaimed powder in cement-stabilized macadam.

## 2. Materials and Experimental Methods

### 2.1. Material

#### 2.1.1. Cement

The cement used in the test was Grade 42.5 ordinary Portland cement, and its technical indicators are listed in Table 1, which meet the requirements specified in Technical Guidelines for Construction of Highway Roadbases (JTG/T F20-2015) [26]. The samples were obtained from Jinhua Cement Plant, Jinhua, Zhejiang, China.

#### 2.1.2. Reclaimed Powders

**Figure 1:**
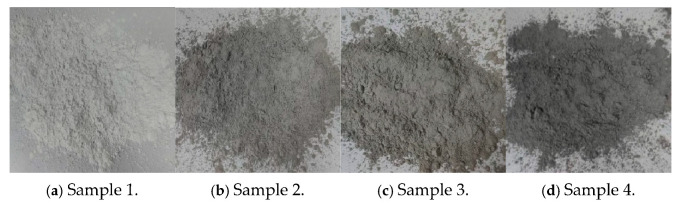
Reclaimed powder samples with different MB values.

By adjusting the mixing ratio of stone powder and mud powder, four types of reclaimed powders with different MB values were prepared, as shown in Figure 1, and their technical indicators are listed in Table 2. Specifically, The samples were obtained from Zhejiang Jinhua Xunda Construction Co., Ltd., Jinhua, Zhejiang, China. Samples 1, 2, 3, and 4 correspond to reclaimed powders with MB values of 0.5, 2.5, 3.5, and 5.0 g/kg, respectively.

#### 2.1.3. Coarse Aggregates

**Figure 2:**
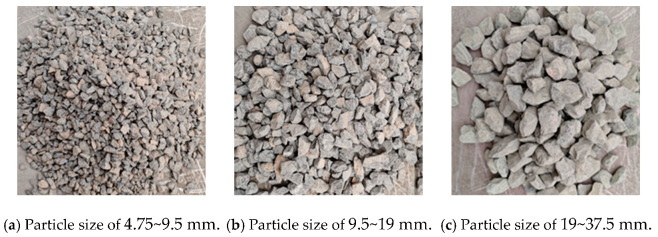
Appearance of coarse aggregates with different specifications.

The coarse aggregates used in the test were limestone with particle sizes of 4.75–9.5 mm, 9.5–19 mm, and 19–37.5 mm. The appearance of the samples is shown in Figure 2, and the technical indicators are listed in Table 3. The samples were obtained from Jinhua Crushing Plant, Jinhua, Zhejiang, China.

#### 2.1.4. Fine Aggregate

**Figure 3:**
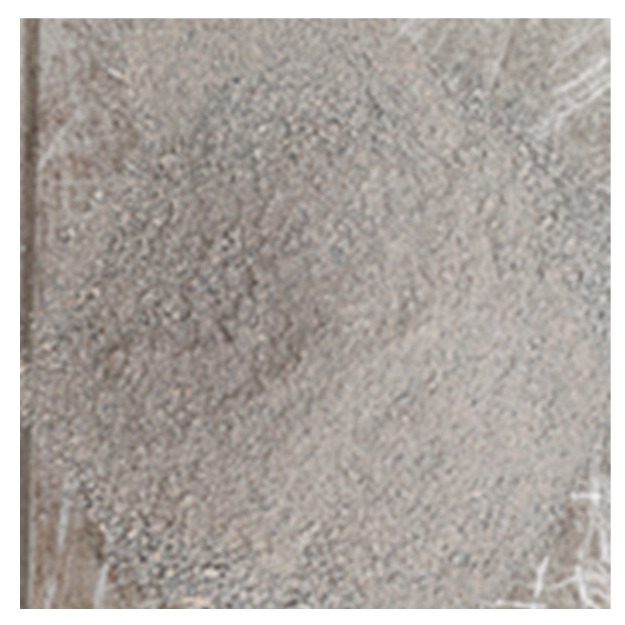
Image of 0~4.75 mm limestone manufactured sand.

The fine aggregate used in the test was 0–4.75 mm limestone manufactured sand. The appearance of the sample is shown in Figure 3, and its technical indicators are listed in Table 4. All performance indicators meet the requirements for fine aggregates used in pavement base courses specified in Technical Guidelines for Construction of Highway Roadbases (JTG/T F20-2015). The samples were obtained from Jinhua Crushing Plant, Jinhua, Zhejiang, China.

### 2.2. Test Design

#### 2.2.1. Mineral Aggregate Gradation

The mineral aggregate gradation is shown in Table 5.

#### 2.2.2. Test Plan

To investigate the influence laws of the MB value, content of reclaimed powder, and cement content on the mechanical strength of cement-stabilized macadam, the following test plan was adopted:

(1) In accordance with Technical Specifications for Design and Construction of Highway Cement Stabilized Macadam Against Cracking (DB 41/T 864-2013), the vertical vibration method was used to form cement-stabilized macadam test specimens with curing ages of 7 days and 28 days. The correlation between the mechanical strength of test specimens formed by the vertical vibration method and that of on-site cement stabilized macadam core samples is as high as 90%, while the correlation of specimens formed by the traditional static compaction method with on-site core samples is less than 70% [27]. Therefore, it can better characterize the vibratory compaction environment of on-site construction.

(2) Compressive strength and splitting strength were used to evaluate the mechanical strength of reclaimed powder and cement-stabilized macadam, both of which were tested in accordance with Test Methods of Materials Stabilized with Inorganic Binders for Highway Engineering (JTG 3441-2024) [28].

(3) We studied the influence laws of the MB value of reclaimed powder, reclaimed powder content, and cement content on the mechanical strength of cement-stabilized macadam. Specifically, the MB values of reclaimed powder were proposed to be 0.5, 2.5, 3.5, and 5.0 g/kg; the reclaimed powder contents were proposed to be 0, 3%, 4%, 5%, 6%, and 7%; and the cement contents were proposed to be 3%, 4%, and 5%.

Since mineral powder is used as the filler in asphalt mixtures, the active stone powder component in reclaimed powder is highly homologous to mineral powder. Based on this characteristic, this study prepared reclaimed powders with different MB values by adjusting the mixing ratio of mineral powder and mud powder.

## 3. Influence of Reclaimed Powder on the Mechanical Strength of Cement-Stabilized Macadam

### 3.1. Influence of Reclaimed Powder on the Compressive Strength of Cement-Stabilized Macadam

#### 3.1.1. Failure Process and Morphology of Specimens

**Figure 4:**
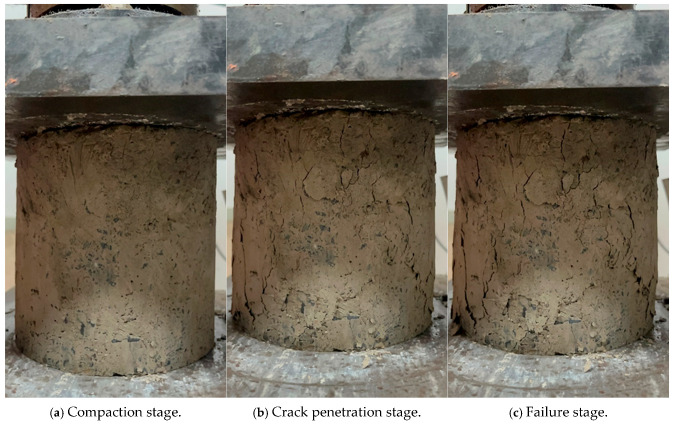
Compressive failure process of cement-stabilized macadam mixed with reclaimed powder.

The failure process of a cement-stabilized macadam specimen mixed with reclaimed powder is shown in Figure 4.

Figure 4a shows the compaction stage of the specimen. When the testing machine applied load to the specimen, the internal voids of the specimen gradually decreased and the material was gradually compacted. At this time, the pressure gauge reading changed slightly and there was no obvious change on the specimen surface. Figure 4b represents the crack penetration stage. As the load continued to increase, internal damage began to occur and develop in the compacted specimen. Multiple fine cracks appeared on the side of the specimen, and these cracks gradually extended, expanded, and connected with each other, with the pressure gauge reading increasing linearly. Figure 4c shows the failure stage. With a further increase in the load, the internal damage of the material accumulated continuously. The cracks on the side of the specimen kept expanding and finally penetrated longitudinally, and the pressure gauge reading stabilized at a certain value, which was the peak stress of the specimen. At this point, the specimen failed, with its middle diameter increasing into a bulging shape. Continued loading led to a rapid drop in the pressure gauge reading, complete penetration of cracks on the specimen surface accompanied by spalling of the binder, and final crushing of the specimen. Observing the uniaxial compression process of specimens with different mix ratios, it was found that the failure stages and morphologies of all specimens were relatively similar.

#### 3.1.2. Influence of the MB Value of Reclaimed Powder

The test results regarding the influence of the MB value of reclaimed powder on the compressive strength of cement-stabilized macadam are shown in Table 6 and Table 7.

As can be seen from the above tables, when the MB value of reclaimed powder is less than 5.0 g/kg, the compressive strength of cement-stabilized macadam decreases slightly with the increase in the MB value of reclaimed powder; when the MB value of reclaimed powder is 5.0 g/kg, compared with 0.5 g/kg, the 7 days compressive strength of cement-stabilized macadam with cement contents of 3%, 4%, and 5% decreases by 2.3–3.5%, 2.2–3.2%, and 1.9–2.8% respectively, and the 28 days compressive strength decreases by 1.8–2.7%, 1.5–2.2%, and 1.3–1.9% respectively. This indicates that when the MB value of reclaimed powder is less than 5.0 g/kg, it has no significant effect on the compressive strength of cement-stabilized macadam.

This is attributed to the comprehensive result of the positive and negative effects of clay powder contained in the reclaimed powder on the compressive strength of cement-stabilized macadam being mutually balanced. On one hand, clay powder can act as a micro-aggregate to fill the small pores and interfacial transition zones of the mixture, thereby improving the compactness and microstructural uniformity of the material, which exerts a certain promotional effect on strength development. On the other hand, clay powder particles tend to adhere to the surface of aggregates to form a coating layer. This not only physically hinders the direct contact between cement paste and aggregates, weakening the interfacial bonding force, but also inhibits the sufficient progress of cement hydration by adsorbing water and occupying reaction space, thereby exerting an adverse effect on strength. Under the conditions of this experiment, the intensities of these two types of action mechanisms were comparable and basically offset each other. Therefore, the MB value of the reclaimed powder did not show a significant trend of influence on the compressive strength overall.

#### 3.1.3. Influence of Reclaimed Powder Content

(1) Variation trend of the influence of reclaimed powder content on the compressive strength of cement-stabilized macadam

**Figure 5:**
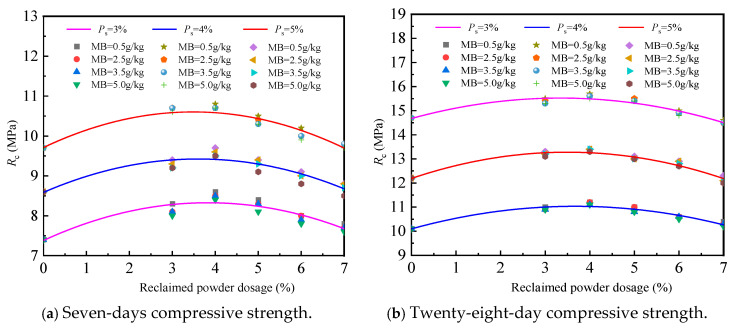
Influence of reclaimed powder content on compressive strength of cement-stabilized macadam.

Based on the data in Table 6 and Table 7, the variation law of the influence of reclaimed powder content on the compressive strength of cement-stabilized macadam is plotted in Figure 5. In the figure, Ps = 3% indicates that the cement content is 3%, and the corresponding curve is the fitted equation curve; the rest cases follow the same rule.

As can be seen from Figure 5, with the increase in reclaimed powder content, the 7 days and 28 days compressive strengths of cement-stabilized macadam both approximately exhibit a parabolic variation law, and the compressive strength reaches the peak value when the reclaimed powder content is 3–4%. The quadratic equation fitting results between the compressive strength of cement-stabilized macadam and the reclaimed powder content are listed in Table 8, with the correlation coefficient reaching 0.85.

(2) Influence of 4% reclaimed powder content on compressive strength

**Figure 6:**
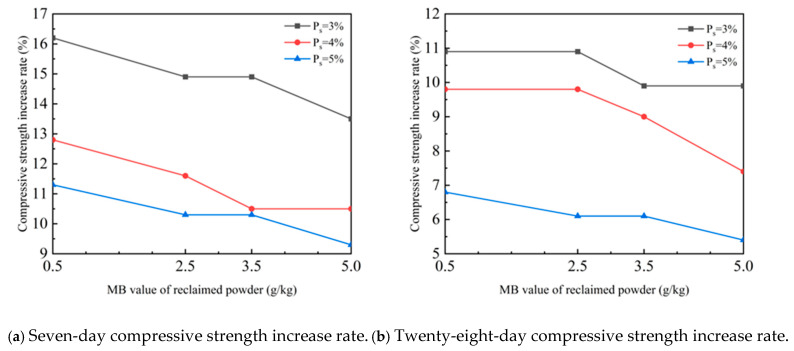
Curve of the influence law of recycled powder with different MB values on the compressive strength improvement rate of cement-stabilized macadam.

The variation curve of the influence of reclaimed powders with different MB values on the compressive strength increase rate of cement-stabilized macadam when the reclaimed powder content is 4% is shown in Figure 6.

As can be seen from Figure 6, when the reclaimed powder content is 4%, compared with the case without reclaimed powder, the 7 days compressive strength of cement-stabilized macadam with reclaimed powder (at cement contents of 3%, 4%, and 5%) increases by 13.5–16.2%, 10.5–12.8%, and 9.3–11.3%, respectively, and the 28 days compressive strength increases by 9.9–10.9%, 9.6–9.9%, and 5.4–6.8%, respectively.

An appropriate dosage of reclaimed powder can effectively fill the gaps between mixture particles through the micro-filling effect and reduce the overall porosity, thereby improving compactness and optimizing the continuity of the microstructure. Meanwhile, the main mineral component of reclaimed powder is calcium carbonate, which possesses certain reactivity. In the later stage of hydration, it can further react with cement hydration products to generate hydrated carboaluminates and promote the formation of ettringite. These secondary hydration products and the micro-filling effect form a synergistic effect, which further clogs and refines internal pores, makes the internal structure of the material more dense, and ultimately synergistically improves its mechanical strength. However, when the content of reclaimed powder is excessively high, its negative effects will dominate: On one hand, an excessive amount of fine particles will deteriorate the mixture gradation, reduce the proportion of coarse aggregates, and weaken the supporting effect of the skeleton structure. On the other hand, excessive reclaimed powder will physically dilute cement hydration products and chemically fail to sustain the strengthening reaction due to limited reactive components, ultimately leading to a decrease in cementing capacity. The combined action of the above two factors results in a reduction in the bulk strength of the mixture and ultimately a decrease in compressive strength.

In summary, when the content of reclaimed powder is 4%, the positive effects of its micro-filling effect and reactivity effect are slightly superior to the negative effects of gradation deterioration and dilution effect, and the comprehensive effect promotes a slight increase in the compressive strength of the material.

### 3.2. Influence of Reclaimed Powder on the Splitting Strength of Cement-Stabilized Macadam

#### 3.2.1. Failure Process and Morphology of Specimens

**Figure 7:**
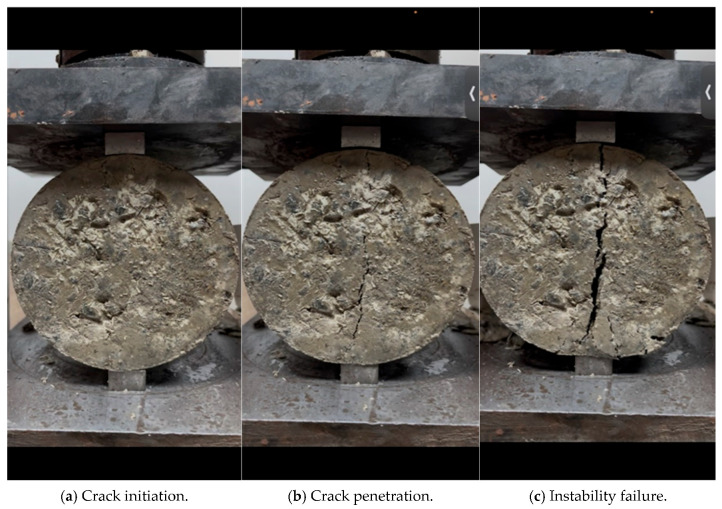
Splitting failure process of cement-stabilized macadam with reclaimed powder.

The failure process of a cement-stabilized macadam specimen mixed with reclaimed powder is shown in Figure 7.

Figure 7a shows the elastic deformation and microcrack initiation stage of the specimen. When the testing machine applied splitting load, radial tensile stress activated the microdefects inside the specimen, and the material mainly underwent elastic deformation. At this time, the testing machine reading rose steadily, with no obvious change on the specimen surface. Figure 7b shows the stage of stable crack propagation and connection. As the load continued to increase, the radial tensile stress exceeded the local bond strength, and microcracks began to initiate in the middle and inside of the specimen’s tension surface. The cracks mainly extended along the aggregate particle interface and gradually connected, with intermittent vertical fine cracks appearing on the specimen’s side. The testing machine reading showed an approximately linear increase. Figure 7c represents the unstable splitting failure stage of the specimen. With further increase in the load, the dominant crack expanded rapidly and penetrated longitudinally along the tension surface. After the testing machine reading reached the peak value (splitting strength), it dropped sharply. Observing the splitting process of specimens with different mix ratios, it was found that the failure stages and morphologies of all specimens were relatively similar.

#### 3.2.2. Influence of the MB Value of Reclaimed Powder

The test results regarding the influence of the MB value of reclaimed powder on the splitting strength of cement-stabilized macadam are shown in Table 9 and Table 10.

As can be seen from the above tables, when the MB value of reclaimed powder is less than 5.0 g/kg, the splitting strength of cement-stabilized macadam decreases slightly with the increase in the MB value of reclaimed powder; when the MB value of reclaimed powder is 5.0 g/kg, compared with 0.5 g/kg, the 7 days splitting strength of cement-stabilized macadam with cement contents of 3%, 4%, and 5% decreases by 3.1–3.4%, 2.4–4.2%, and 1.9–4.0%, respectively, and the 28 days splitting strength decreases by 2.0–3.0%, 1.7–3.4%, and 1.4–2.1%, respectively. This indicates that when the MB value of reclaimed powder is less than 5.0 g/kg, it has no significant effect on the splitting strength of cement-stabilized macadam.

When the MB value of reclaimed powder is less than 5.0 g/kg, its influence mechanism on the splitting strength of cement-stabilized macadam is similar to that of compressive strength. The mud powder also exerts a micro-filling effect, so it does not directly cause defects such as pores and cracks inside the mixture; however, the mud powder first adheres to the surface of the aggregate, which not only hinders the bonding between cement paste and aggregate, thereby reducing the interface strength between aggregate and cement hydration products, but also inhibits the cement hydration reaction, ultimately leading to a decrease in splitting strength.

#### 3.2.3. Influence of Reclaimed Powder Content

(1) Variation trend of the influence of reclaimed powder content on the splitting strength of cement-stabilized macadam

**Figure 8:**
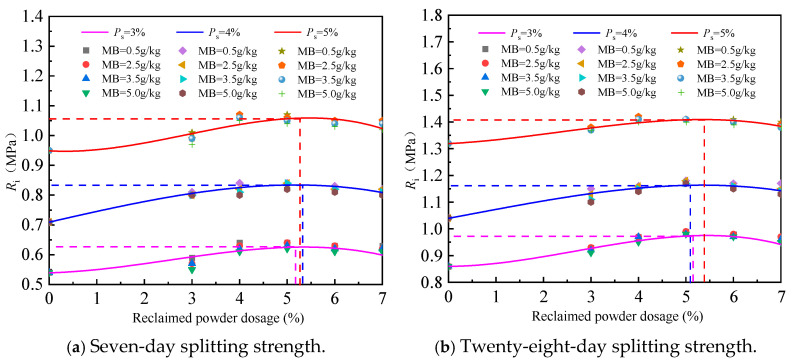
Influence curves of reclaimed powder content on splitting tensile strength of cement-stabilized macadam.

Based on the data in Table 9 and Table 10, the curves of the variation law of the influence of reclaimed powder content on the splitting strength of cement-stabilized macadam are plotted in Figure 8. In the figure, Ps = 3% indicates that the cement content is 3%, and the corresponding curve is the fitted equation curve; the rest cases follow the same rule.

As can be seen from Figure 8, with the increase in reclaimed powder content, the 7 days and 28 days splitting strengths of cement-stabilized macadam first increase and then decrease, and the splitting strength reaches the peak value when the reclaimed powder content is 5–6%. The cubic equation fitting results between the splitting strength of cement-stabilized macadam and the reclaimed powder content are listed in Table 11, with the correlation coefficient reaching 0.80.

(2) Influence of 4% reclaimed powder content on splitting strength

**Figure 9:**
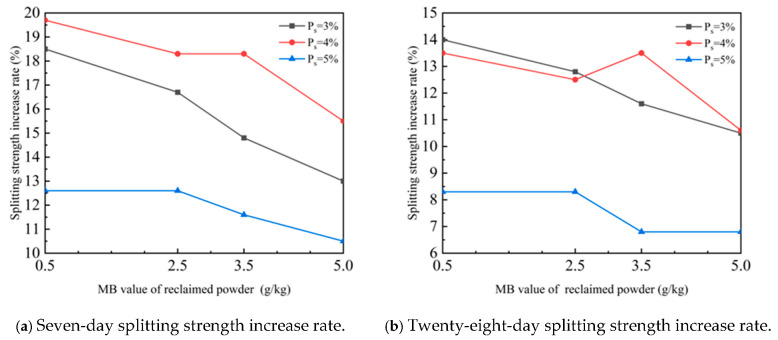
Curve of the influence law of recycled powder with different MB values on the splitting strength improvement rate of cement-stabilized macadam.

The curve of the variation law of the influence of reclaimed powders with different MB values on the splitting strength increase rate of cement-stabilized macadam when the reclaimed powder content is 4% is plotted in Figure 9.

As can be seen from Figure 9, when the reclaimed powder content is 4%, compared with the case without reclaimed powder, the 7 days splitting strength of cement-stabilized macadam with reclaimed powder (at cement contents of 3%, 4%, and 5%) increases by 13.0–18.5%, 15.5–18.3%, and 10.5–12.6%, respectively, and the 28 days splitting strength increases by 10.5–14.0%, 12.5–13.5%, and 6.8–8.3%, respectively.

Splitting strength directly reflects the interfacial bonding performance between aggregates and cement paste, thus being particularly sensitive to microscopic defects (e.g., pores, microcracks) inside the mixture. An appropriate dosage of reclaimed powder effectively fills the tiny pores and interfacial transition zones within the mixture through its micro-filling effect, thereby reducing the overall porosity and improving compactness. This not only optimizes the continuity of the internal structure of the material but also reduces stress concentration points, enabling more uniform load transfer. Therefore, within the range of content not exceeding 4%, the splitting strength shows a rapid growth trend. However, when the reclaimed powder content exceeds the appropriate range, its negative effects begin to dominate, which is specifically manifested in two aspects: On one hand, an excessive amount of fine particles hinders the normal hydration process of cement; due to physical dilution and water competition, the strength development of the overall cementitious system is insufficient. On the other hand, excessive reclaimed powder tends to accumulate in local areas to form weak zones, which not only reduces the uniformity of the material but also forms a physical barrier between aggregates and paste, directly weakening the bonding strength between them. Consequently, macroscopically, the splitting strength decreases with the increase in reclaimed powder content.

Considering the influence of reclaimed powder content on the compressive strength and splitting strength of cement-stabilized macadam comprehensively, the optimal content of reclaimed powder should be controlled at around 4%, with a maximum not exceeding 5%.

## 4. Equivalence Analysis of Reclaimed Powder and Cement Content

**Figure 10:**
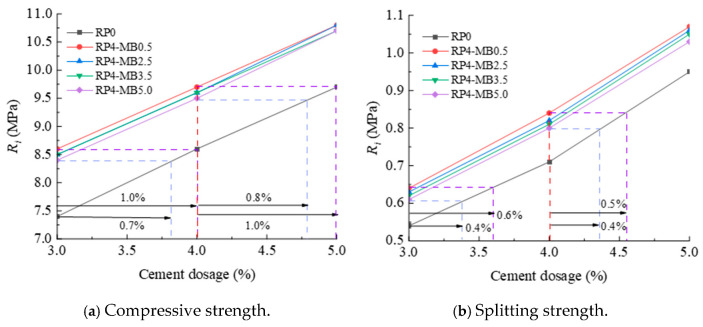
Influence curves of cement content on mechanical strength of cement-stabilized macadam.

The 7-day compressive strength and splitting strength are key indicators for determining whether cement-stabilized macadam meets the strength design requirements. Therefore, based on the principle of 7 days strength equivalence, the variation law of the influence of cement content on the mechanical properties of different types of cement-stabilized macadam is plotted in Figure 10. In the figure, RP4-MB0.5 indicates the external addition of 4% reclaimed powder with an MB value of 0.5 g/kg, and the rest of the expressions follow the same rule.

The following can be seen from Figure 10:

(1) With the increase in cement content, the compressive strength and splitting strength of cement-stabilized macadam without reclaimed powder and with 4% reclaimed powder of various MB values all approximately show a linear increasing trend.

(2) Within the range of the reclaimed powder’s MB value of 0.5–5.0 g/kg, the 7 days compressive strength and 7 days splitting strength of cement-stabilized macadam with 4% reclaimed powder and 3% cement content are equivalent to those of cement-stabilized macadam without reclaimed powder with 3.8–4.0% and 3.4–3.6% cement content, respectively.

(3) Within the range of the reclaimed powder’s MB value of 0.5–5.0 g/kg, the 7 days compressive strength and 7 days splitting strength of cement-stabilized macadam with 4% reclaimed powder and 4% cement content are equivalent to those of cement-stabilized macadam without reclaimed powder with 4.8–5.0% and 4.4–4.5% cement content, respectively.

In summary, when the cement content is 3% or 4% and the reclaimed powder content is 4%, based on the principle of equivalent compressive strength and splitting strength, respectively, reclaimed powder can replace 0.8–1.0% or 0.4–0.6% of the cement content.

## 5. Comprehensive Benefit Analysis

### 5.1. Economic Benefits

When the reclaimed powder content is 4%, the cement dosage can be reduced by 0.4% to 0.6%, corresponding to a cement saving of 40 to 60 tons per 10,000 tons of cement-stabilized macadam. Based on the national market price of P.O 42.5 grade cement at CYN 358 per ton in December 2025, the direct economic benefits generated by cement saving are CNY 136,000, CNY 152,000, and CNY 168,000 per 10,000 cubic meters of C30, C40, and C50 concrete, respectively. For every 10,000 tons of cement-stabilized macadam, the direct economic benefit can reach CNY 14,000 to 21,000. Meanwhile, 400 tons of reclaimed powder can be absorbed per 10,000 tons of cement-stabilized macadam. Calculated at a landfill disposal cost of CNY 300 per ton, an additional disposal cost saving of CNY 152,000 can be achieved, further enhancing the economic feasibility of the technology application.

### 5.2. Social and Environmental Benefits

The production of 1 ton of cement consumes an average of approximately 200 kg of coal and 100 kWh of electricity, accompanied by 0.85 tons of CO_2_ emissions. By saving 40 to 60 tons of cement per 10,000 tons of cement-stabilized macadam, CO_2_ emissions can be reduced by 34.5 to 51.6 tons, lowering the carbon emission intensity of cement production. Furthermore, the combustion of coal during cement production releases toxic and harmful gases such as CO, SO_2_, and NO_x_, and the alternative application of reclaimed powder can indirectly reduce the emission of such pollutants. From the perspective of reclaimed powder disposal, its output is large but the resource utilization rate is relatively low. Except for a small amount used as filler in asphalt mixtures, most of it is disposed of through stacking or landfilling. During stacking disposal, particles with a particle size of ≤0.075 mm are prone to scattering under wind action, causing air pollution and threatening the health of surrounding populations. Landfill disposal not only occupies limited land resources but also may lead to groundwater pollution as reclaimed powder seeps into the ground after being washed by rainwater.

In summary, the resourceful application of reclaimed powder in cement-stabilized macadam can not only save cement consumption and reduce resource depletion and pollutant emissions during cement production but also realize the efficient disposal of reclaimed powder, solving the problems of severe environmental pollution and high disposal costs. It boasts significant environmental and social benefits.

## 6. Conclusions

This study investigated the influence laws of the MB value and content of reclaimed powder on the compressive strength and splitting strength of cement-stabilized macadam, established fitting equations for the compressive strength and splitting strength with respect to the reclaimed powder content; proposed the required MB value and recommended content of reclaimed powder; explored the influence law of the reclaimed powder’s MB value on mechanical strength under this content condition; and analyzed the equivalence between reclaimed powder and cement content based on the principle of consistent mechanical strength. The main conclusions are as follows:

(1) Within the MB value range of the reclaimed powder adopted in the test (≤5.0 g/kg), the mechanical strength of cement-stabilized macadam decreases slightly with the increase in the reclaimed powder’s MB value, but the maximum reduction amplitude does not exceed 4.2%.

(2) When the reclaimed powder content is ≤7%, the mechanical strength of cement-stabilized macadam shows a trend of first increasing and then decreasing with the increase in the reclaimed powder content. The compressive strength and splitting strength reach their peak values when the reclaimed powder content is 3.0–4.0% and 5.0–5.5%, respectively.

(3) Compared with the case without reclaimed powder, the 7 days compressive strength and splitting strength of cement-stabilized macadam with 4% reclaimed powder can be increased by at least 9.3% and 10.5%, respectively, and the 28 days compressive strength and splitting strength can be increased by at least 5.4% and 6.8%, respectively. The addition of reclaimed powder can significantly improve the strength of cement-stabilized macadam and is more conducive to the formation of early strength.

(4) With higher cement content, the improvement effect of reclaimed powder on the mechanical strength of cement-stabilized macadam gradually becomes weaker. Considering mechanical strength and economic benefits comprehensively, when the cement content is 3–4%, adding 4% reclaimed powder can replace at least 0.4–0.5% of the cement content.

## Figures and Tables

**Table 1:** Technical specifications of cement.

Setting Time (min)		Compressive Strength (MPa)		Flexural Strength (MPa)	
Initial	Final	3 days	28 days	3 days	28 days
173	219	30.4	50.5	5.1	7.5

**Table 2:** Technical specifications of reclaimed powder.

Sample	Density (g/cm^3^)	Hydrophilic Coefficient	MB Value (g/kg)
Sample 1	2.76	0.64	0.5
Sample 2	2.61	0.76	2.5
Sample 3	2.63	0.78	3.5
Sample 4	2.57	0.84	5.0

**Table 3:** Technical specifications of coarse aggregates.

Particle Size (mm)	Apparent Relative Density	Flaky and Elongated Particle Content (%)	Water Absorption (%)	Crushing Value (%)	Abrasion Value (%)	Soundness (%)	Soft Stone Content (%)
4.75–9.5	2.729	--	1.69	17.9	18.9	3.8	1.4
9.5–19	2.731	12.9	0.79				
19–37.5	2.756	7.5	0.59				

**Table 4:** Technical specifications of fine aggregate.

Apparent Density (g/cm^3^)	Clay Lump Content (%)	Soundness (%)	Crushing Value (%)
2.728	0.0	7.1	16

**Table 5:** Gradation of mineral aggregate.

Sieve Aperture (mm)	37.5	31.5	19.0	9.5	4.75	2.36	0.6	0.075
**Mass passing percentage (%)**	100.0	99.4	60.0	41.7	30.7	22.8	10.1	2.0

**Table 6:** Test results of influence of MB value of reclaimed powder on 7 days compressive strength of cement-stabilized macadam.

Cement Content (%)	Reclaimed Powder Content (%)	Compressive Strength of Cement-Stabilized Macadam with Reclaimed Powders of Different MB Values (MPa)			
		0.5 g/kg	2.5 g/kg	3.5 g/kg	5.0 g/kg
3	0	7.4	7.4	7.4	7.4
	3	8.3	8.1	8.1	8.0
	4	8.6	8.5	8.5	8.4
	5	8.4	8.3	8.3	8.1
	6	8.0	8.0	7.9	7.8
	7	7.8	7.7	7.7	7.6
4	0	8.6	8.6	8.6	8.6
	3	9.4	9.3	9.2	9.2
	4	9.7	9.6	9.5	9.5
	5	9.4	9.4	9.3	9.1
	6	9.1	9.0	9.0	8.8
	7	8.8	8.8	8.7	8.5
5	0	9.7	9.7	9.7	9.7
	3	10.7	10.6	10.6	10.4
	4	10.8	10.7	10.7	10.6
	5	10.5	10.4	10.3	10.3
	6	10.2	10.0	10.0	9.9
	7	9.8	9.8	9.8	9.7

**Table 7:** Test results of influence of MB value of reclaimed powder on 28 days compressive strength of cement-stabilized macadam.

Cement Content (%)	Reclaimed Powder Content (%)	Compressive Strength of Cement-Stabilized Macadam with Reclaimed Powders of Different MB Values (MPa)			
		0.5 g/kg	2.5 g/kg	3.5 g/kg	0.5 g/kg
3	0	10.1	10.1	10.1	10.1
	3	11.0	10.9	10.9	10.9
	4	11.2	11.2	11.1	11.1
	5	11.0	11.0	10.8	10.8
	6	10.6	10.6	10.6	10.5
	7	10.4	10.3	10.3	10.2
4	0	12.2	12.2	12.2	12.2
	3	13.3	13.2	13.2	13.1
	4	13.4	13.4	13.3	13.1
	5	13.1	13.0	13.0	13.0
	6	12.9	12.9	12.8	12.7
	7	12.3	12.1	12.1	12.0
5	0	14.7	14.7	14.7	14.7
	3	15.5	15.4	15.3	15.2
	4	15.7	15.6	15.6	15.5
	5	15.5	15.5	15.4	15.3
	6	15.0	14.9	14.9	14.8
	7	14.6	14.5	14.5	14.4

**Table 8:** Fitting equations between compressive strength of cement-stabilized macadam and reclaimed powder content.

Curing Age (Days)	Cement Content (%)	Fitting Equation	*R* ^2^	Reclaimed Powder Content Corresponding to Peak Strength (%)
7	3	$y=0.49x-0.06x^{2}+7.38$	0.871	3.82
	4	$y=0.47x-0.06x^{2}+8.59$	0.865	3.60
	5	$y=0.51x-0.07x^{2}+9.71$	0.920	3.48
28	3	$y=0.51x-0.07x^{2}+10.10$	0.940	3.67
	4	$y=0.62x-0.09x^{2}+12.19$	0.952	3.50
	5	$y=0.51x-0.08x^{2}+14.67$	0.907	3.34

**Table 9:** Test results of influence of MB value of reclaimed powder on 7 days splitting tensile strength of cement-stabilized macadam.

Cement Content (%)	Reclaimed Powder Content (%)	Splitting Strength of Cement-Stabilized Macadam with Reclaimed Powders of Different mb Values (MPa)			
		0.5 g/kg	2.5 g/kg	3.5 g/kg	5.0 g/kg
3	0	0.54	0.54	0.54	0.54
	3	0.59	0.58	0.59	0.57
	4	0.64	0.63	0.62	0.61
	5	0.64	0.64	0.63	0.62
	6	0.62	0.61	0.60	0.59
	7	0.62	0.60	0.61	0.59
4	0	0.71	0.71	0.71	0.71
	3	0.81	0.80	0.80	0.78
	4	0.85	0.84	0.84	0.82
	5	0.84	0.84	0.84	0.82
	6	0.83	0.82	0.82	0.81
	7	0.82	0.82	0.81	0.80
5	0	0.95	0.95	0.95	0.95
	3	1.01	0.99	0.99	0.97
	4	1.07	1.07	1.06	1.05
	5	1.07	1.06	1.05	1.04
	6	1.05	1.05	1.04	1.02
	7	1.04	1.04	1.02	1.02

**Table 10:** Test results of influence of MB value of reclaimed powder on 28 days splitting tensile strength of cement-stabilized macadam.

Cement Content (%)	Reclaimed Powder Content (%)	Splitting Strength of Cement-Stabilized Macadam with Reclaimed Powders of Different mb Values (MPa)			
		0.5 g/kg	2.5 g/kg	3.5 g/kg	5.0 g/kg
3	0	0.86	0.86	0.86	0.86
	3	0.93	0.92	0.92	0.91
	4	0.98	0.97	0.96	0.95
	5	0.99	0.97	0.98	0.96
	6	0.97	0.96	0.97	0.95
	7	0.95	0.95	0.94	0.94
4	0	1.04	1.04	1.04	1.04
	3	1.15	1.13	1.11	1.10
	4	1.18	1.17	1.18	1.15
	5	1.17	1.16	1.15	1.14
	6	1.16	1.16	1.16	1.15
	7	1.16	1.15	1.14	1.13
5	0	1.32	1.32	1.32	1.32
	3	1.38	1.37	1.36	1.35
	4	1.43	1.43	1.41	1.41
	5	1.41	1.41	1.40	1.38
	6	1.41	1.40	1.40	1.39
	7	1.40	1.39	1.38	1.38

**Table 11:** Fitting equations between splitting tensile strength of cement-stabilized macadam and content of reclaimed powder.

Curing Age (d)	Cement Content (%)	Fitting Equation	*R* ^2^	Reclaimed Powder Content Corresponding to Peak Strength (%)
7	3	$y=-0.0098x^{3}+0.0074x^{2}+0.0044x+0.5392$	0.813	5.21
	4	$y=-0.0004x^{3}-0.0002x^{2}+0.0366x+0.7094$	0.936	5.36
	5	$y=-0.0017x^{3}+0.0144x^{2}-0.0090x+0.9488$	0.814	5.32
28	3	$y=-0.0014x^{3}+0.0113x^{2}+0.0012x+0.8595$	0.935	5.43
	4	$y=-0.0005x^{3}+0.0006x^{2}+0.0332x+1.0394$	0.896	5.12
	5	$y=-0.0009x^{3}+0.0061x^{2}+0.0091x+1.3192$	0.810	5.17

## Data Availability

The original contributions presented in this study are included in the article. Further inquiries can be directed to the corresponding author.

## References

[1] Esquinas A.R., Álvarez J.I., Jiménez J.R., Fernández J.M., De Brito J. (2018). Durability of Self-Compacting Concrete Made with Recovery Filler from Hot-Mix Asphalt Plants. Constr. Build. Mater..

[2] Valentin J. (2021). Characterization of Quarry Dusts and Industrial By-Products as Potential Substitutes for Traditional Fillers and Their Impact on Water Susceptibility of Asphalt Concrete. Constr. Build. Mater..

[3] Parmar N., Kondraivendhan B., Khungar H. (2025). Experimental and Analytical Investigation of Metakaolin and Quarry Dust in Concrete. Suranaree J. Sci. Technol..

[4] Shi Y., Huo J., Wang Y., Lin Y., Deng Q., Peng S. (2025). Mechanistic Investigation of Machine-Made Sand Methylene Blue Value Effects on Mortar Performance. Appl. Sci..

[5] Kufre Etim R., Ufot Ekpo D., Christopher Attah I., Chibuzor Onyelowe K. (2021). Effect of Micro Sized Quarry Dust Particle on the Compaction and Strength Properties of Cement Stabilized Lateritic Soil. Clean. Mater..

[6] Abdalqader A., Sonebi M., Thornton N., Taylor S. (2022). Factorial Design Modelling of Cement Grout Containing Dolomitic Quarry Dust Powder. Mater. Today Proc..

[7] Jain D., Gupta R., Choudhary R., Alomayri T., Agrawal V. (2022). Utilization of Marble Dust and Fly Ash in Composite Mortar as Partial Cement Substitute. Mater. Today Proc..

[8] Umar M., Qian H., Khan M.N.A., Siddique M.S., Almujibah H., Elshekh A.E.A., Bashir M.O., Vatin N.I. (2025). Strength and Durability of Concrete with Bentonite Clay and Quarry Dust. Front. Mater..

[9] Nair D.G., Thomas R.V. (2023). Quarry Dust as a Fine Aggregate Replacement in Concrete Masonry Blocks for Sustainable Construction. Int. J. Sustain. Constr. Eng. Technol..

[10] Nakayenga J., Cikmit A.A., Tsuchida T., Hata T. (2021). Influence of Stone Powder Content and Particle Size on the Strength of Cement-Treated Clay. Constr. Build. Mater..

[11] Sundaralingam K., Peiris A., Anburuvel A., Sathiparan N. (2022). Quarry Dust as River Sand Replacement in Cement Masonry Blocks: Effect on Mechanical and Durability Characteristics. Materialia.

[12] Cohen E., Peled A., Bar-Nes G. (2019). Dolomite-Based Quarry-Dust as a Substitute for Fly-Ash Geopolymers and Cement Pastes. J. Clean. Prod..

[13] Chitkeshwar A.K., Naktode P.L. (2022). Concrete with Rock Quarry Dust with Partial Replacement of Fine Aggregate. Mater. Today Proc..

[14] Alizada A.M., Waseem E., Kudus S.A., Jaini Z.M. (2023). Optimisation of Ultra-High-Performance Concrete with Special Quarry Dust. Int. J. Integr. Eng..

[15] Nakayenga J., Inui M., Guharay A., Hata T. (2023). Effect of Limestone and Granite Stone Powder on Properties of Cement-Treated Clay Composites and Their Socioeconomic and Environmental Impacts. Constr. Build. Mater..

[16] Pakkiyachandran M., Sathiparan N. (2025). Comparative Study on Quarry Waste, Manufactured Sand, Quarry Dust as River Sand Replacement in Cement Mortar: Mechanical Characteristics, Durability, and Eco-Benefit. Materialia.

[17] Borçato A.G., Thiesen M., Medeiros-Junior R.A. (2023). Mechanical Properties of Metakaolin-Based Geopolymers Modified with Different Contents of Quarry Dust Waste. Constr. Build. Mater..

[18] Yuan Q., Wang L., Kong D., Han Y., Ren C., Tian Y., Zhu G. (2024). Synergistic Action and Effect Mechanism of Coal Gangue Powder and Red Mud on the Properties of Concretes. J. Build. Eng..

[19] Ngugi J., Rading G.O., Mbuya T.O. (2025). Characterisation of Quarry Dust for Use as a Filler Material. MRS Adv..

[20] Li S., Chen D., Jia Z., Li Y., Li P., Yu B., Li S., Chen D., Jia Z., Li Y. (2023). Effects of Mud Content on the Setting Time and Mechanical Properties of Alkali-Activated Slag Mortar. Materials.

[21] Zhao H., Ma Y., Zhang J., Hu Z., Li H., Wang Y., Liu J., Wang K. (2022). Effect of Clay Content on Shrinkage of Cementitious Materials. Constr. Build. Mater..

[22] (2022). Sand for Construction.

[23] (2018). Concrete for Railway Construction.

[24] Zhou H., Ge C., Chen Y., Song X. (2023). Study on Performance and Fractal Characteristics of High-Strength Manufactured Sand Concrete with Different MB Values. Front. Earth Sci..

[25] Bentur A., Larianovsky P., Bentur A., Larianovsky P. (2024). Marginal Aggregates: The Role of Clays. Materials.

[26] (2015). Technical Guidelines for Construction of Highway Roadbases.

[27] Yi Y., Jiang Y., Tian T., Fan J., Deng C., Xue J. (2022). Mechanical-Strength-Growth Law and Predictive Model for Ultra-Large Size Cement-Stabilized Macadam Based on the Vertical Vibration Compaction Method. Constr. Build. Mater..

[28] (2024). Test Methods of Materials Stabilized with Inorganic Binders for Highway Engineering.

